# From Biomimicking to Bioinspired Design of Electrocatalysts for CO_2_ Reduction to C_1_ Products

**DOI:** 10.1002/anie.202314446

**Published:** 2023-10-16

**Authors:** Panagiotis Trogadas, Linlin Xu, Marc‐Olivier Coppens

**Affiliations:** ^1^ EPSRC “Frontier Engineering” Centre for Nature Inspired Engineering Department of Chemical Engineering University College London Torrington Place London WC1E 7JE United Kingdom

**Keywords:** Bio-Inspired, Bio-Mimicking, C_1_ Products, CO_2_ Reduction, Electrocatalysts

## Abstract

The electrochemical reduction of CO_2_ (CO_2_RR) is a promising approach to maintain a carbon cycle balance and produce value‐added chemicals. However, CO_2_RR technology is far from mature, since the conventional CO_2_RR electrocatalysts suffer from low activity (leading to currents <10 mA cm^−2^ in an H‐cell), stability (<120 h), and selectivity. Hence, they cannot meet the requirements for commercial applications (>200 mA cm^−2^, >8000 h, >90 % selectivity). Significant improvements are possible by taking inspiration from nature, considering biological organisms that efficiently catalyze the CO_2_ to various products. In this minireview, we present recent examples of enzyme‐inspired and enzyme‐mimicking CO_2_RR electrocatalysts enabling the production of C_1_ products with high faradaic efficiency (FE). At present, these designs do not typically follow a methodical approach, but rather focus on isolated features of biological systems. To achieve disruptive change, we advocate a systematic design methodology that leverages fundamental mechanisms associated with desired properties in nature and adapts them to the context of engineering applications.

## Graphical abstract

A nature‐inspired chemical engineering (NICE) approach for the design of nature‐inspired electrocatalysts for the reduction of CO_2_ to C_1_ products is presented in this Minireview.

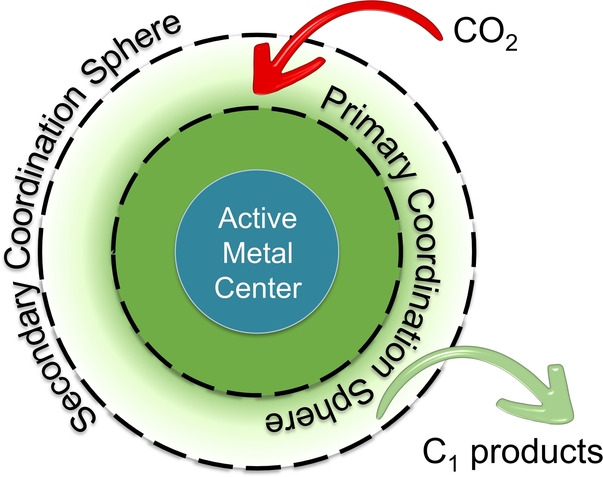

## 1 Introduction

### 1.1 Fundamentals of CO_2_ Reduction

**Figure 1:**
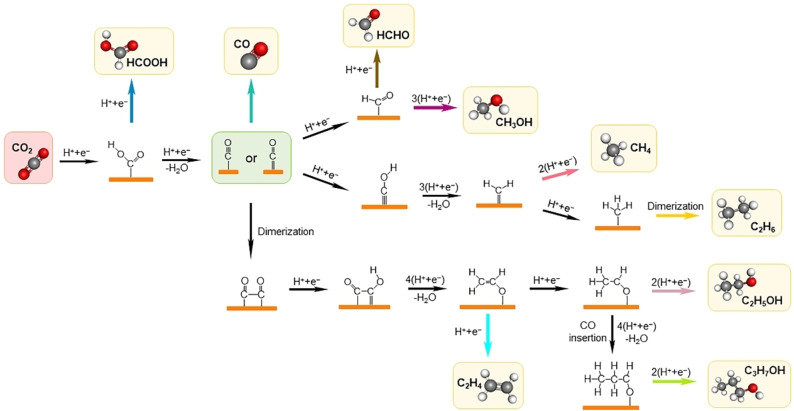
Overview of possible CO_2_ reduction pathways for C_1_ and C_2+_ products.

Electrocatalytic reduction of CO_2_ (CO_2_RR) offers a compelling avenue to address growing concerns about our carbon footprint, as it offsets carbon emissions and produces value‐added products.^[1]^ It is a multiple proton and electron transfer reaction (Eq. 1) resulting in the formation of several products and water (Figure 1)

(1) $$kCO_{2}+n\left(H^{+}+e^{-}\right)↔Product+mH_{2}O$$

where *k*, *n*, and *m* are the reaction coefficients (e.g., *k*=1, *n*=2, *m*=1 when Product=CO; *k*=2, *n*=12, *m*=3, when Product=CH_3_CH_2_OH, etc.).^[2]^ If the reduction of CO_2_ involves the transfer of two electrons and protons (*n*=2), then the overall reaction is reversible, since the employed electrocatalyst can catalyze the corresponding reactions in both directions. On the contrary, if CO_2_RR occurs via the transfer of more electrons and protons (*n*>2), then additional intermediates are formed and the overall reaction becomes irreversible, as the binding energy of each intermediate follows linear scaling relationships.^[2]^ These scaling relationships are due to the presence of similar chemical bonds between adsorbed species and catalytic surfaces imposing a high overpotential for the reduction of CO_2_. Examples include the scaling relationship between *OCHCH_2_, *OCHCH_3_, and *OCH_2_CH_3_ intermediates for the reduction of CO_2_ to CH_3_CH_2_OH, *OH and *OCHCH_2_ intermediates for the reduction of CO_2_ to C_2_H_4_, etc.^[2]^

### 1.2 Electrocatalyst Design: The Heart of The Challenge

Several noble (Au, Ag) and non‐noble (Cu, Ni, Bi, Fe, Mn, etc.) metals, alloys, metal oxides, and transition metal chalcogenides are employed as CO_2_RR electrocatalysts.^[3]^ Among them, copper is the most widely used electrocatalyst, since it can directly reduce CO_2_ to hydrocarbons and alcohols, such as methane, ethanol, CO, and formate.^[4]^ However, Cu based electrocatalysts exhibit poor selectivity and large overpotentials, due to the complexity of the reaction pathway for hydrocarbon production involving several proton and electron transfer steps.^[4]^

Noble metal based electrocatalysts (such as Au, Pd, Ag, etc.) have also been used for the reduction of CO_2_ to CO and formate.^[5]^ Even though these electrocatalysts exhibit high selectivity towards the production of CO (FE_CO_>90 % for Au and Ag based electrocatalysts)^[6]^ and formate (FE_formate_>97 % for Pd based electrocatalysts),^[7]^ their high cost and scarcity makes them less attractive for large scale production.

Metal nitrogen doped carbon (MNCs) materials are a promising alternative to these electrocatalysts due to their stability and high selectivity toward CO_2_RR. Ni based MNCs are the most efficient electrocatalysts to produce CO (FE_CO_ >90 %) due to the low binding energy of Ni toward H* suppressing the hydrogen evolution (HER) reaction.^[4a]^ Further research is needed to improve these materials, as the origin of their activity is not well understood. Thus far, a very low FE_CH4_ (≈0.4 %)^[8]^ to produce CH_4_ is achieved due to the inability of MNCs to co‐adsorb CO* and H* limiting CO protonation.^[9]^ Detailed review about CO_2_RR electrocatalysts in general is beyond the scope of this article and the reader is referred to recent, thorough reports in the literature for additional information.[^3^, ^4^, ^5^, ^8^]

From these reviews, it becomes clear that the lack of rational design principles for the development of highly efficient and selective CO_2_RR catalysts impedes disruptive progress in CO_2_RR devices. Key challenges facing current electrocatalysts are their low selectivity towards desired products and their durability, since a very limited number of electrocatalysts reported thus far can exceed 120 h of continuous CO_2_RR without a significant loss in their activity.[^1^, ^3a^, ^3d^, ^10^] Additionally, they should mediate multiple proton and electron transfers to CO_2_ without resorting to excessive reducing overpotentials, leading to low energy efficiency.^[11]^ Finally, it is crucial to embrace additional factors affecting their activity and selectivity, such as the local environment around the active sites (concentration of available CO_2_) and the structure of the catalyst layer, which can influence the transport of reacting species.^[12]^

In terms of local environment, the local pH at the interface between the catalyst and the electrolyte can significantly affect the selectivity of the electrocatalyst, since the overall reaction rate depends on the selected pH value (Eq. 2).^[13]^ The local pH is different from the pH of the bulk electrolyte due to the enhanced formation rate of OH^−^ during CO_2_RR and HER reactions, which increases the pH near the surface of the electrocatalyst.[^4a^, ^14^]
(2)

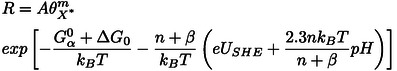




where *R* is the overall reaction rate, *A* is the reaction prefactor, 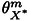 is the coverage of species X*, $G_{\alpha }^{0}$ is the activation energy of the process at 0 V vs. standard hydrogen electrode (SHE), $\Delta G_{0}$ is the free energy of the process at 0 V vs. SHE, *U_SHE_* is the potential vs. SHE, *e* is the electric elementary charge of an electron, *n* is the number of proton‐electron transfers before the rate‐limiting step, *β* is the transfer coefficient, *k*_B_ is the Boltzmann constant, and *T* is the reaction temperature. For *n*=0, the reaction rate depends only on U_SHE_.^[13]^

The shift in pH values ($-2.3k_{B}T\Delta \left(pH\right)\frac{\beta }{e\left(n+\beta \right)}$ ) creates different overpotentials favoring the formation of different products. A≈−71 mV and ≈−357 mV shift in overpotential for C_1_ and C_2_ products, respectively, between pH=7 and pH=13 is reported in the case of Cu electrocatalysts.^[13]^ The observed ≈0.36 V shift in overpotential for C_2_ products represents a two‐orders of magnitude increase in C_2_ selectivity compared to C_1_ products in that pH range.^[13]^ The C_2_ production is limited by the rate of first proton‐electron transfer to the O=C−C=O intermediate (Figure 1) and CO coverage at low and high overpotentials, respectively.^[13]^ On the contrary, C_1_ production is limited by the rate of proton‐electron transfer to the CHOH intermediate, resulting in a smaller increase in activity with increasing pH, compared to C_2_ formation.^[13]^

A similar trend is observed for noble metal based electrocatalysts to produce CO; the selectivity of Ag electrocatalysts (FE_CO_ >85 %) is enhanced at local pH values greater than the buffer pH (≈7),^[15]^ while for Au electrocatalysts, CO selectivity does not depend on local pH as it is limited by the CO_2_ adsorption step.^[16]^ For MNCs, pH plays an important role in their selectivity as well. High faradaic efficiency (>80 %) toward CO production is achieved for Fe−N−C electrocatalysts at high pH values (≥7), where the HER is suppressed.^[17]^

**Figure 2:**
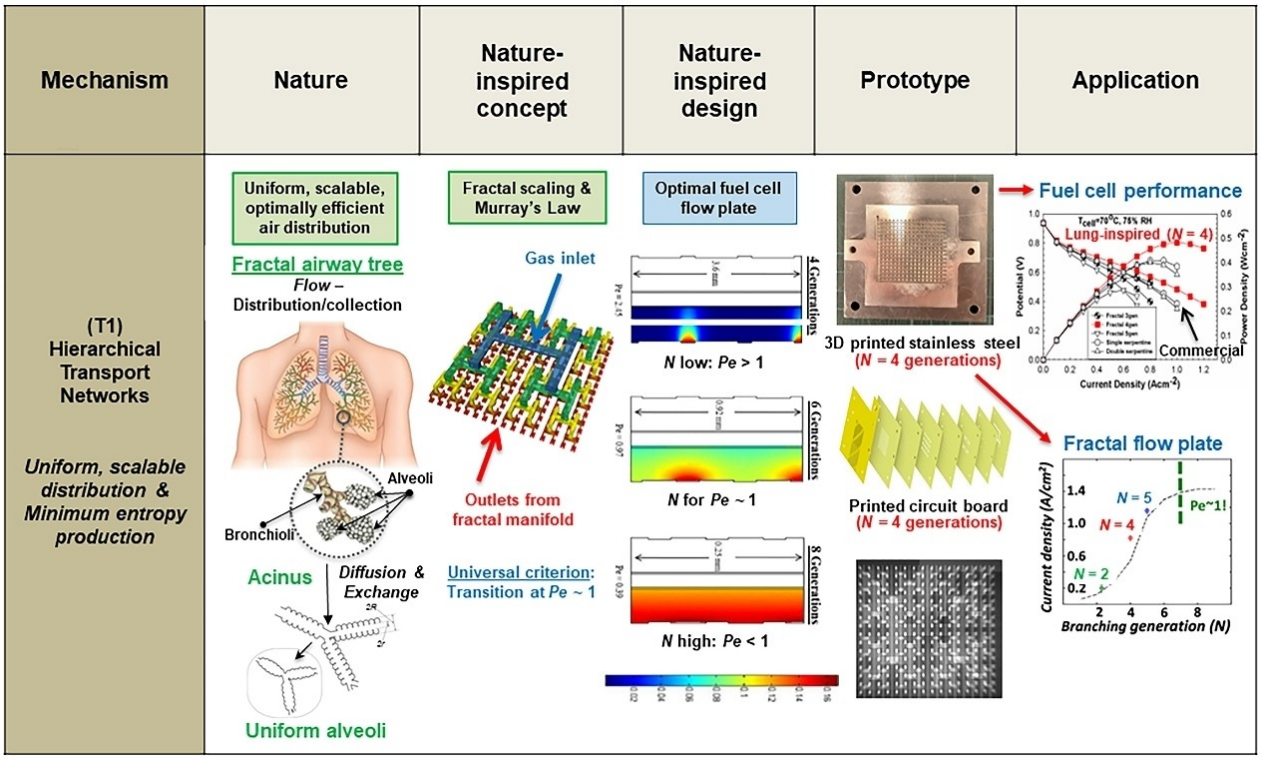
Systematic employment of the NICE methodology for the design and engineering of lung‐inspired flow fields for proton exchange membrane fuel cells (PEMFCs). This figure is adapted from ref. [19].

Based on the above challenges, it is evident that a novel approach to design CO_2_RR electrocatalysts could drive accelerated innovation. Incremental changes through minor design modifications or alteration of the reaction conditions may not produce the required transformative solutions. An example of such approach is our nature‐inspired chemical engineering (NICE) methodology^[18]^ (discussed in more detail in Section 4) which recognizes universal fundamental mechanisms in nature underpinning desired properties (like scalability, efficiency, and resilience), which can be leveraged to achieve similar properties in an applied context. These ubiquitous mechanisms define NICE Themes, of which there are presently four: (T1) hierarchical transport networks; (T2) force balancing and nano‐confinement; (T3) dynamic self‐organization; (T4) ecosystems, networks, and modularity. For example, (T1) includes ubiquitous networks that greatly promote reliably scalable performance across a wide range of length scales, as observed in trees, lungs, and the vascular network. Within or across these themes, NICE deploys a design methodology that comprises four stages to bridge nature with technology in applications, namely: nature‐inspired concept, design, prototype, and application (Figure 2). Figure 2 presents an example of the application of the NICE methodology to design lung‐inspired flow fields for proton exchange membrane fuel cells (PEMFCs).^[19]^ Inspiration is derived from the lung (“Nature”) due to its ability to scale‐up irrespective of size, providing uniform distribution of oxygen into the blood stream while keeping the thermodynamic losses across its volume at a minimum. This is achieved via its fractal architecture, ensuring that the Péclet number, Pé, is close to 1, and, hence, the convective air flow dominating the upper part of the lung is equal to the diffusive air flow at the lower part of the lung (“Nature‐inspired concept”). A mathematical model is built based on these characteristics of the lung to calculate the optimum number of fractal generations in a flow field to achieve Pé ≈1 (“Nature‐inspired design”), and then, lung‐inspired flow fields are created via 3D printing (“Prototype”) exhibiting higher performance (≈30 % increase in current and power density) and ≈75 % lower pressure drop than serpentine flow field based PEMFCs (“Application”) minimizing the parasitic power losses.^[19]^

In terms of CO_2_RR electrocatalysts, Nature can be an excellent guide to rational design, as it is full of biological organisms that efficiently catalyze the same reactions as the electrocatalysts and robust structures that are intrinsically scaling. Based on recent literature, we now review opportunities resulting from imitating or taking inspiration from nature to design better catalysts. We focus on C_1_ products, since they are important chemical feedstocks to produce fuels and value‐added chemicals.

## 2 Biomimetic Electrocatalysts for CO_2_ Reduction to C_1_ Products

The most widely used source of inspiration for the design of novel electrocatalysts for CO_2_RR are metalloenzymes, which have exceptional catalytic efficiency and selectivity towards the same reactions occurring in electrochemical CO_2_ reduction devices. Biomimetic design is focused on isolated features of biological organisms (such as their chemical structure), whereas bioinspired design considers both the structure and function of metalloenzymes recognizing the different context between the biological example and the technological application.

### 2.1 Enzyme‐Mimicking Electrocatalysts

A successful biomimetic design approach is based on the presence of imidazole groups of histidine residues at the active site of carbon monoxide dehydrogenases (CODHs) facilitating proton transfer to and from the active site via the formation of hydrogen bonds with water molecules.^[20]^

As a result, N,N‐di(2‐picolyl)ethylenediamine (DPEN), a source of imidazole groups, is incorporated into iron porphyrin, one of the most active and selective electrocatalysts for CO_2_ to CO conversion in organic solvents,^[21]^ and its properties are evaluated towards CO_2_RR in acetonitrile. The poly‐pyridine/amine sites of DPEN form hydrogen bonds with water molecules assisting in proton transfer, as they function as multiple proton relays, and the protonated DPEN units are positively charged, stabilizing the negatively charged CO_2_RR reduction intermediates.^[22]^ Hence, the iron porphyrin with incorporated DPEN units is highly active towards CO_2_RR, exhibiting a TOF of ≈5 ⋅ 10^4^ s^−1^ (acetonitrile (MeCN) electrolyte solution) for CO_2_ to CO conversion with water as the proton source, four times higher than the activity of DPEN‐free iron porphyrin.^[22]^

Another efficient biomimetic electrocatalyst is pentlandite (Fe_4.5_Ni_4.5_S_8_), due to its structural resemblance with CODHs, as it contains Fe−Ni sites with a bond length of ≈2.6 Å, similar to the ones in CODHs (≈2.8 Å), connected via sulfur atoms.^[23]^ The efficiency of CO_2_RR depends on the concentration of protons (the water content in the chosen solvent to conduct CO_2_RR) at the surface of the electrocatalyst. The selectivity of the electrocatalyst towards CO_2_RR increases as the water content decreases (MeCN electrolyte solution), and an ≈87 % FE for CO is observed at low water concentration (≈24 ppm H_2_O).^[23]^ This preliminary result illustrates the potential of aprotic solvents with low water content as electrolytes for CO_2_RR.^[23]^

### 2.2 Alveolus‐Mimicking Electrocatalysts

**Figure 3:**
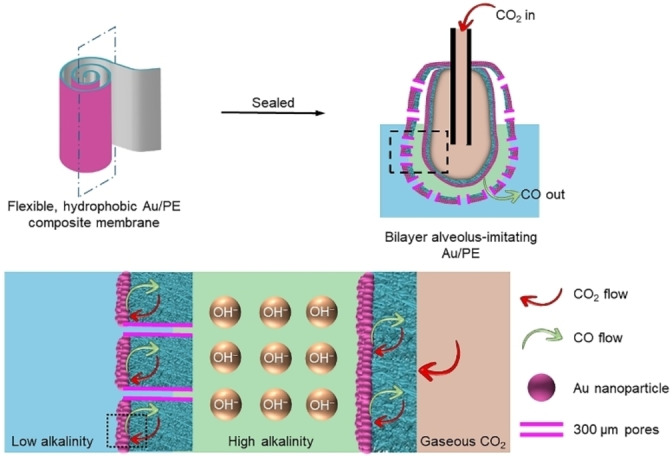
Alveolus‐mimicking electrocatalyst consisting of a bilayer porous and hydrophobic Au/PE composite membrane. This figure is adapted from ref. [26].

The efficiency of CO_2_RR electrocatalysts can also be improved via an increase of the local CO_2_ concentration. The ratio of CO_2_ to H_2_O molecules in an aqueous solution is ≈1 : 1,300 at 1 atm pressure; this CO_2_ concentration can be increased by increasing the pressure,^[24]^ but this is only a temporary solution. To design such electrocatalyst with high gas permeability and low water diffusivity, the structure of the mammalian lung is imitated, where the alveoli are enclosed by several epithelial membranes (≈1 μm thickness) with high gas permeability and low water diffusivity.^[25]^ Hence, an artificial alveolus is engineered from a flexible polyethylene (PE) membrane sputtered with a thin layer (≈20 nm thickness) of gold nanoparticles (Figure 3). The PE membrane is hydrophobic and porous (pore radius ≈40–500 nm) making it impermeable to water but allowing gas transport. Gold serves as the catalyst in this study as it is highly efficient towards the production of CO.^[26]^ This Au/PE composite membrane is rolled into a bilayer structure, and its bottom and top edges are sealed to form a closed pouch‐type structure. The activity of this biomimicking electrocatalyst is evaluated in an H‐type cell achieving ≈92 % Faradaic efficiency of CO production (in a CO_2_‐saturated 0.5 M potassium bicarbonate (KHCO_3_) electrolyte solution).^[26]^

## 3 Bioinspired Electrocatalysts for CO_2_ Reduction to C_1_ Products

Enzyme‐inspired electrocatalysts derive their inspiration more broadly from the protein scaffold surrounding the active metal center of metalloenzymes, which is responsible for their high activity and selectivity. Their structural characteristic is the presence of primary and secondary (or outer) coordination spheres, which contribute significantly to the function of metalloenzymes. The primary coordination sphere is dominated by covalent interactions between ligands and metal ions; the number of ligands and the oxidation state of the metal affect spin‐state ordering and reactivity.^[27]^ The secondary coordination sphere represents the residues that do not directly bind to the active metal center but interact with the primary ligands via long‐range interactions, modulating the catalytic properties of the metalloenzymes. These long‐range interactions, such as hydrogen‐bonding interactions or a salt‐bridge to the substrate or the primary ligands, charge stabilization of a nearby anion or cation binding site, alter the redox potential of the metal, local charge distribution, and electron transfer, tuning the catalytic behavior of the metal center.[^27^, ^28^]

### 3.1 Dehydrogenase‐Inspired Electrocatalysts

In nature, the enzyme dehydrogenase (such as CODH, formate DH, alcohol DH, etc.) catalyzes the selective reversible conversion of CO_2_ to C_1_ and C_2+_ products via hydrogen bonding interactions between the bound substrate and the amino acid residues present in the secondary coordination sphere.^[20a]^ However, the direct application (“bio‐integration”) of DHases in electrochemical devices is impractical, since they have oxygen intolerance, resulting in low stability and current density.^[20a]^ Thus, to circumvent these issues, the engineering of electrocatalysts inspired by the structure of these enzymes is required.

Mo−Cu and Ni−Fe are commonly used as the active metal centers of these bio‐inspired electrocatalysts for the reduction of CO_2_ to C_1_ products (formate and methane, respectively).^[29]^ For Mo−Cu based bio‐inspired electrocatalysts, a [(bdt)Mo^VI^(O)S_2_Cu^I^CN]^2−^ complex is used in which Mo and Cu ions are connected via sulfide and benzenedithiolate (bdt) ligands resembling the carbon monoxide dehydrogenase enzyme (CODH2).^[29a]^ The reduction (*n*=2 e^−^) of [(bdt)Mo^VI^(O)S_2_Cu^I^CN]^2−^ forms CO_3_^2−^ and the complex [(bdt)Mo^IV^S_2_Cu^I^CN]^2−^ whose protonation creates Mo^V^H hydride intermediates that react with CO_2_ to form formate, as revealed by infrared spectroelectrochemical (IR‐SEC) studies coupled with density functional theory (DFT) computations.^[29a]^ This Mo−Cu based bio‐inspired electrocatalyst exhibits good selectivity for formate over CO (9 : 1) but low activity (turnover number=4 in 0.1 M trifluoroethanol (TFE) electrolyte solution), demonstrating that further fundamental research is required to comprehend the interplay between the two metal centers.^[29a]^

For Ni−Fe based bio‐inspired electrocatalysts, a [L^N2S2^Ni^II^Fe^II^Cp(CO)]^+^ (L^N2S2^=2,2‐(2,2‐bipryridine‐6,6‐diyl)bis(1,1‐diphenylethanethiolate)) complex is used to resemble NiFe dehydrogenase enzyme.^[29c]^ It exhibits high activity (TON_CH4_=3.5 ⋅ 10^5^ and TON_H2_=8 ⋅ 10^6^ at pH=4) and its selectivity is affected by the pH value as the H_2_/CH_4_ product ratio fluctuates between 50 : 1 and 30 : 1 at pH=3 and 5, respectively.^[29c]^ The reaction mechanism is still unknown for this Ni−Fe complex; it is speculated that CO_2_ is activated and reduced at one metal site, while the second metal site delivers hydride to the first metal site until CH_4_ is produced.^[29c]^

**Figure 4:**
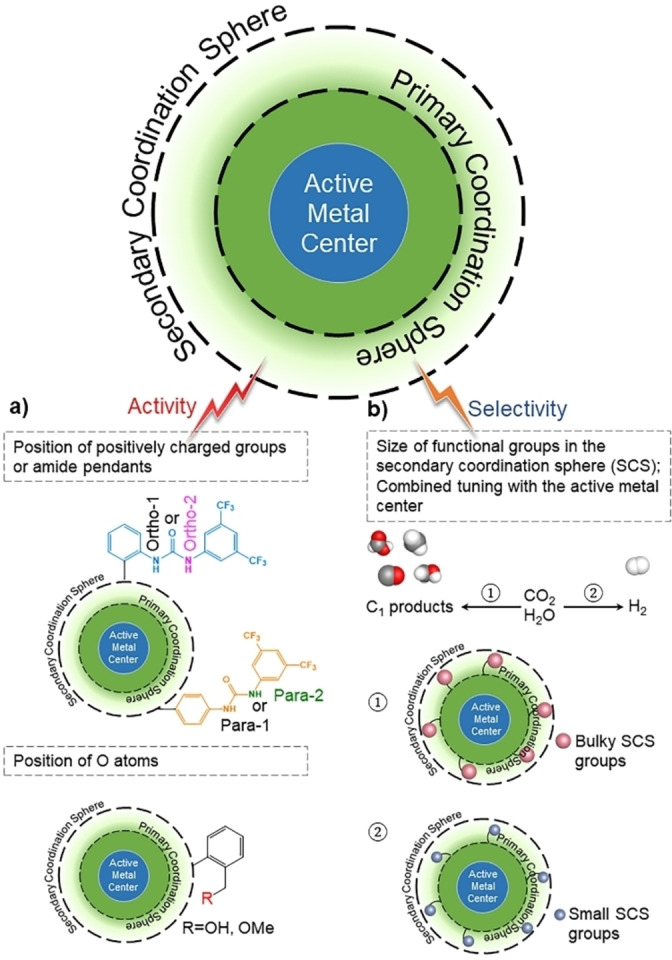
Tuning the activity (a) and selectivity (b) of dehydrogenase‐inspired CO_2_RR electrocatalysts.

Thus, the design of such electrocatalysts is extremely challenging, as there are several intertwined factors affecting their activity and selectivity. Their activity is also influenced by the presence of O atoms, and the position of a positively charged group or amide pendants in the outer coordination sphere, while their selectivity is influenced by the size of functional groups in the secondary coordination sphere (Figure 4a and b).

To investigate the effect of the position of a positively charged group (such as arginine, histidine, or lysine) in the outer coordination sphere on CO_2_ hydrogenation to formate, several artificial enzymes have been synthesized as variants of the active catalyst Rh‐LmrR (Rh‐bisdiphosphine complex [Rh(PN^glycine^P)_2_]^−^ covalently incorporated into a 119‐residue protein (LmrR‐lactoccal multidrug resistant regulator).^[28a]^ Proton NMR measurements reveal that the D100 position is influential in catalysis, with the D100R mutant exhibiting a three‐fold increase in catalytic activity compared to Rh‐LmrR (≈0.7 and 0.2 h^−1^ TOF, respectively).^[28a]^ In D100R mutant, aspartic acid is replaced by arginines, which are placed near the active metal center; this positively charged group attracts CO_2_, increasing its concentration near the metal center and, hence, enhancing the reaction rate. Even though the position of a positively charged group affects the activity of these artificial enzymes, there are several additional factors that must be considered as well: the accurate positioning of the charged group may be inhibited due to the presence of hydrogen bonds or salt bridges, which prohibit the incorporation of residues near the active metal center and, thus, do not increase the reaction rate. Water distribution around the active metal center may influence the catalytic activity too, according to molecular dynamic simulations. Preliminary results demonstrate that the population of water around the most active electrocatalyst D100R is significantly reduced compared to the other electrocatalysts, which may enhance its activity.^[28a]^

To examine the effect of the position of amide pendants in the secondary coordination sphere on CO_2_RR to CO (Figure 4a), iron tetraphenylporphyrin (Fe‐TPP) derivatives bearing amide pendants at various positions (ortho‐1, −2, and para‐1, −2) at the periphery of the metal core are investigated.^[30]^ Fe‐TPP derivatives with proximal and distal amide pendants on the ortho‐ position of the phenyl ring demonstrate significantly higher TOF_CO_ values compared to their counterparts with amide pendants on the para‐position (TOF_CO_ ≈5.5 ⋅ 10^6^ s^−1^ and ≈6.8 ⋅ 10^3^ s^−1^ for Fe‐ortho‐2‐amide and Fe‐para‐2‐amide, respectively using 0.1 M tetrabutylammonium hexafluorophosphate (TBAPF_6_) in dimethylformamide (DMF) as electrolyte solution).^[30]^ Fe‐ortho‐2‐amide has the smallest H−O distance (≈1.45 Å) or largest through‐space interactions, indicating that properly positioned amide pendants increase the affinity for CO_2_ via hydrogen bonding interactions.^[30]^

Apart from amide pendants, amine pendants (NH−R) or phenolic groups can be introduced in the ortho positions of the phenyl ring resulting in ≈98 % and ≈90 % FE_CO_, respectively (0.1 M tetrabutylammonium tetrafluoroborate (n‐Bu_4_NPF_4_) in DMF as electrolyte solution).^[31]^ The former increase is due to the presence of amine pendants decreasing the overpotential of CO_2_ reduction,^[31a]^ while the latter enhancement in FE is due to the high local concentration of protons associated with the phenolic groups.^[31b]^

The presence of O atoms in the secondary coordination sphere (Figure 4a) also plays a role in the activity of the catalyst towards CO_2_RR.^[32]^ Oxymethyl‐ether (−OMe) and hydroxyl (−OH) pendant groups are incorporated into the outer coordination sphere of iron‐bipyridine compounds and their activity towards CO_2_RR to formate is examined. The presence of these pendant groups in the secondary coordination sphere modifies the protonation reaction of iron‐bound O atoms; the excess O atoms enhance the net proton transfer to the active site increasing the production of formate in the presence of −OMe (FE_HCOO_≈85 %) and −OH (FE_HCOO_≈71 %) groups, respectively, compared to conventional iron‐bipyridine complex (FE_HCOO_≈68 %) using 0.1 M TBAPF_6_ in DMF as electrolyte solution.^[32a]^ Hence, the utilization of moieties in the secondary coordination sphere to improve metal‐ligand interactions and proton transfer to the active metal center is a powerful strategy for tuning the activity and selectivity of the electrocatalyst.^[32a]^

In terms of the selectivity of the bio‐inspired electrocatalyst (Figure 4b), the size of the functional groups^[33]^ and their combined tuning with the active metal center^[34]^ are crucial parameters.

Small pyridyl‐based groups promote selective H_2_ evolution, since the transfer of hydride to protons is facile, resulting in a ≈78 % FE for H_2_ production.^[33b]^ The active metal center of this bio‐inspired electrocatalyst is [Fe_4_N(CO)_11_L]^−^ where the parent iron cluster [Fe_4_N(CO)_12_]^−^ is an effective electrocatalyst for the selective reduction of CO_2_ to formate (≈95 % FE) at pH=7 and L represents the secondary coordination sphere groups containing phosphine ligands functionalized with aprotic functional groups of different sizes.^[33b]^ On the contrary, large N,N‐dimethylamine groups support selective formation of formate, as they hinder protonation of the active site and selectivity for hydride transfer to CO_2_ is increased, resulting in the production of formate with ≈70 % FE (0.1 M n‐Bu_4_NBF_4_ in MeCN/H_2_O (95 : 5) as electrolyte solution).^[33b]^

The incorporation of bipyridine‐modified ligands, in which two benzylic amines are positioned in the secondary coordination sphere of the active metal center can alter the selectivity of the electrocatalyst towards specific products of CO_2_ reduction. These benzylic amines serve as proton transfer relays and form ([Metal]‐H) units, which, in turn, create “formato” compounds ([Metal]‐O_2_CH) upon interaction with CO_2_, resulting in the formation of formic acid (HCOOH). If the active metal center is rhenium (Re) or ruthenium (Ru), the binding of CO_2_ onto the metal is more favorable than the formation of ([Re/Ru]‐H) and it exhibits high selectivity towards CO production (FE_CO_≈80 % in a 0.2 M Bu_4_NBF_4_ in MeCN electrolyte solution).^[34a]^ On the contrary, if Mn serves as the active metal center, then the formation of ([Mn]‐H) compound is more favorable than the binding of CO_2_ onto the metal and it results in high selectivity towards the production of HCOOH (FE_HCOOH_≈80 %, TOF >4000 s^−1^, 0.2 M Bu_4_NBF_4_ in MeCN electrolyte solution).^[34]^

### 3.2 Dehydrogenase‐Inspired Electrocatalysts with Pseudo‐Secondary Coordination Sphere

Apart from the utilization of different ligands for the formation of a secondary coordination sphere, another successful strategy is the employment of cationic buffers (such as imidazole, bicarbonate, phosphate, and triethanolamine) replacing the long‐chain ligands in the outer coordination sphere of DHases. In this catalyst design, [Ni(cyclam)]^2+^ (cyclam=1,4,8,11‐tetraazacyclotetradecane) is the active metal center which catalyzes the reduction of CO_2_ to CO or formate via a proton‐coupled, ECEC (electron transfer‐chemical step‐electron transfer‐chemical step) pathway; its activity and selectivity are increased via pyridine‐ or imidazole‐binding.^[35]^ Imidazole preferentially binds to the Ni^II^ of [Ni(cyclam)]^2+^, stabilizes the divalent oxidation state, and decreases the required reduction potential. Once [Ni(cyclam)]^2+^ is reduced, imidazole buffer plays the role of histidine ligands in DHases, transferring protons to and from the bound substrate, and hence, acting as a pseudo‐secondary coordination sphere.^[35]^

**Figure 5:**
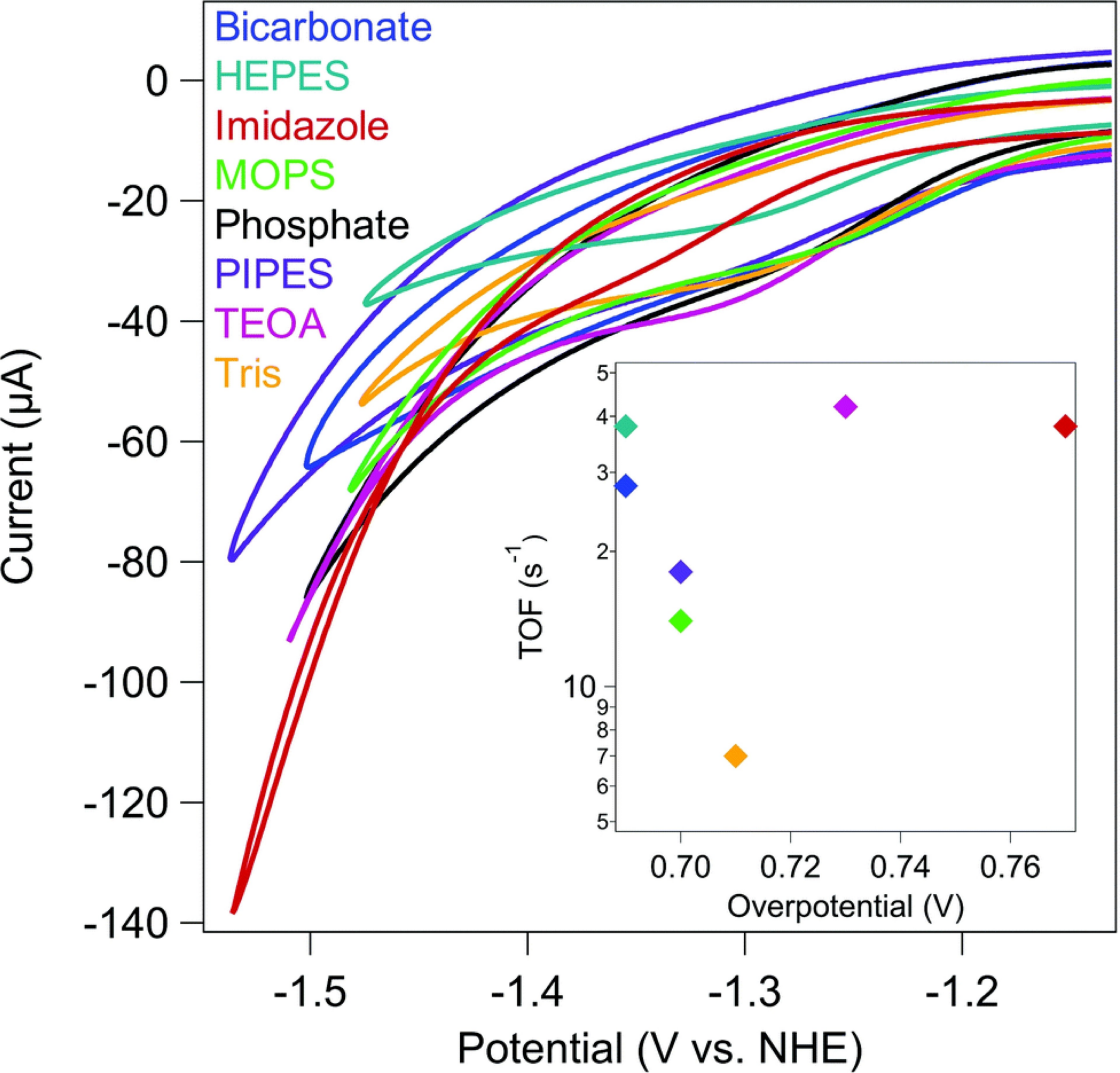
Cyclic voltammograms (ν=1 V s^−1^) of [Ni(cyclam)]^2+^ under CO_2_‐saturated atmosphere. All reactions contained 100 μM [Ni(cyclam)]^2+^, 100 mM KCl, and 100 mM buffer at pH=7. (Inset) TOF as a function of overpotential for each buffer. HEPES is 4‐(2‐hydroxyethyl)‐1‐piperazineethanesulfonic acid, MOPS is 3‐(N‐morpholino)propanesulfonic acid, PIPES is piperazine‐N,N′‐bis(2‐ethanesulfonic acid), TEOA is triethanolamine, and Tris is tris(hydroxymethyl)aminomethane. [Reproduced from ref. [35b] with permission from the Royal Society of Chemistry].

The highest turnover frequencies (TOF_CO_) of ≈50 s^−1^ (100 μM [Ni(cyclam)]^2+^ with 100 mM potassium chloride (KCl) as electrolyte solution) are observed for imidazole (red diamond, Figure 5) and imidazole‐derived buffers (purple diamond, piperazine, Figure 5). However, imidazole buffer exhibits the highest negative electrocatalytic overpotential for CO_2_ reduction (≈0.77 V vs. NHE), compared to bicarbonate buffer (blue diamond) demonstrating the least negative electrocatalytic potential of ≈0.69 V (vs. NHE), similar to the value for the reduction of CO_2_ to CO at pH=7.^[35b]^

## 4 Application of NICE Methodology

The above examples do not explicitly use a systematic design framework, as the one provided by NICE, but instead represent a set of isolated inspirations from natural systems.

Our NICE methodology can address this issue, as it deploys a thematic approach that comprises four stages to bridge nature with technology in applications, namely: nature‐inspired concept, design, and prototyping for experimental realization (Figure 2).

First, the **nature‐inspired concept**, is identified as a mechanism found commonly in nature, which is the crucial ingredient to solve an issue in nature (e.g., the properties of the hierarchical transport network leading to scalability, within T1) that is also pertinent in the envisioned technological application, despite the often different contexts of nature and technology (e.g., a lung and a fuel cell).^[19]^

Second, the **nature‐inspired design** stage translates this concept into a design that is specifically formulated for the intended application; hence, an (abstract) mechanism is adopted but its (concrete) realization needs to be adapted for technological use (e.g., the self‐similar branching of the lung and the dimensioning of its channels for scalable, minimum entropy production, need to be adapted in the context of the different environment and production requirements of a hydrogen fuel cell flow plate, even though both require scalable, maximally efficient air distribution).

Finally, experimental realization is achieved by manufacturing **prototypes** based on the nature‐inspired, often computationally assisted design through experimental testing and characterization to investigate performance under technologically relevant conditions. Prototyping is an iterative process that embraces new synthesis and (e.g., digital and additive) manufacturing techniques.

**Figure 6:**
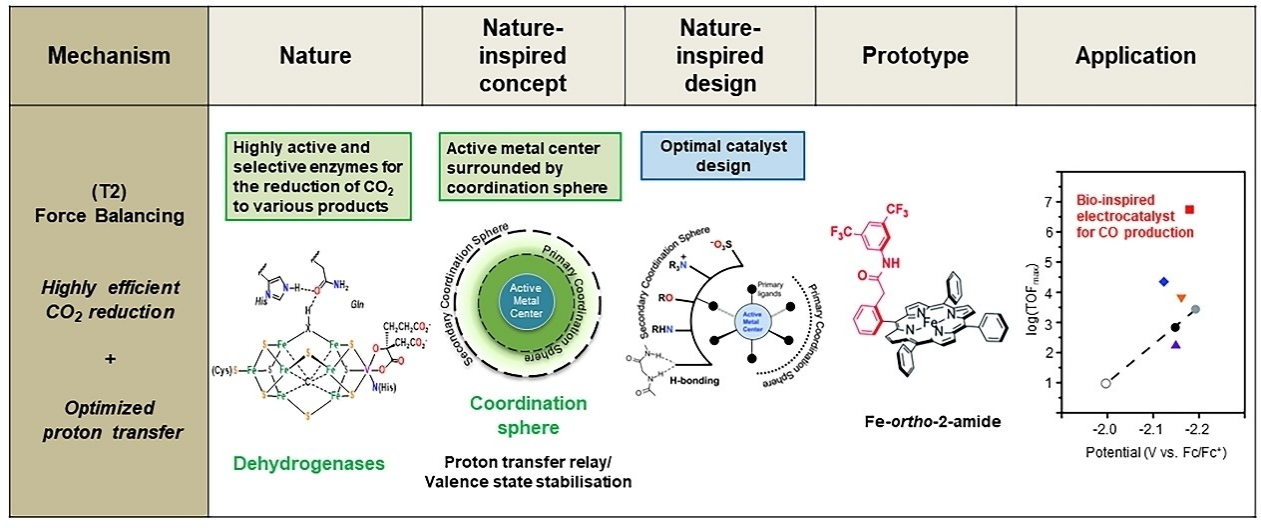
Systematic methodology for the design of nature‐inspired CO_2_RR electrocatalysts. The unit of TOF_max_ values is s^−1^. The figures under “Prototype” and “Application” are reproduced from ref. [30] with permission from the Royal Society of Chemistry.

The design of bio‐inspired CO_2_RR electrocatalysts reported in the literature relates to the T2 theme of NICE methodology (Section 1.2.), namely force balancing, achieved through nano‐confinement. The employment of a secondary sphere to modulate active metal center‐ligand interactions or forces is indeed a successful strategy (Section 3.1.) for the enhancement of their catalytic activity and selectivity (Figure 6).

Are there any additional elements in the design space that could further improve the properties of bioinspired CO_2_RR electrocatalysts? NICE could be employed as an innovation accelerator for the design and development of such materials by following each stage of its methodology, and recognising universal aspects captured by the NICE Themes. This allows us to avoid fragmentation in case‐by‐case catalyst developments.

For the **nature‐inspired concept**, it should be considered that themes T1 (hierarchical transport networks) and T2 (force balancing) harmonically co‐exist in natural systems; in particular, enzymes that catalyze the same reactions as CO_2_RR electrocatalysts rely on both their coordination sphere and hierarchical transport network to obtain their appealing catalytic properties.^[18b]^ The unique structural characteristic of enzymes is the position of the active metal center deep into a substrate channel to effectively separate it from the solution environment.^[18b]^ This structural geometry provides control over the chemical environment where the reaction occurs and the transport of products to new active sites for cascade reactions (substrate channeling). A recent example is an ORR electrocatalyst (PtNi) with similar structure to metalloenzymes:^[36]^ isolated substrate channels are formed between the surface and the center of the nanoparticles, while their exterior surface is passivated by a surfactant to ensure that the electrochemical reactions take place in nanoconfined substrate channels. As a result, this bio‐inspired PtNi electrocatalyst demonstrates a two‐fold increase in ORR activity (acidic media) compared to mesoporous PtNi nanoparticles.^[36a]^ The diameter of their substrate channels greatly impacts the activity of these bio‐inspired PtNi nanoparticles. In the kinetically limited regime where electron transfer dominates (low overpotential), the reaction occurs along the substrate channel and, hence, the smaller its diameter (<≈1.5 nm), the higher the activity of the electrocatalyst.^[36b]^ On the contrary, at high overpotentials where the reaction is mass transport limited, nanoconfinement does not affect the activity of the electrocatalyst, since the reaction occurs at the entrance of the substrate channel, and, thus, the larger its diameter (>2 nm) or accessible electrochemically active area, the higher its activity.^[36b]^

Thus, an electrocatalyst consisting of an optimal combination of nanochannels (between 1–4 nm diameter) to leverage nano‐confinement, a hierarchical transport network to minimize transport limitations,^[37]^ and a coordination sphere to improve the properties of its active metal center could be highly active and selective towards CO_2_RR. Computationally assisted models (**nature‐inspired design**), a step that is often neglected in the reported literature, should be developed first to aid in the design of bio‐inspired electrocatalysts. Molecular modelling could help fundamental understanding to achieve customized selectivity through cascade reactions, lowering the free energy barrier of CO_2_RR and, hence, its overpotential.[^1^, ^38^] Synthesis procedures should then be carefully chosen to create these CO_2_RR electrocatalysts and evaluate their activity, stability, and selectivity in a CO_2_RR device (**prototyping** for **applications**).

## 5 Conclusions and Outlook

In summary, nature is an ideal source of inspiration for the design of artificial CO_2_RR electrocatalysts, as there are plenty of biological examples that efficiently catalyze the same reactions and have robust structures, deployed at scale. The examples presented in Sections 2 and 3 demonstrate that bioinspired design prevails over narrow biomimetics (or bio‐imitation), since it considers the structure and function of the biological example and the different context between nature and technological applications. However, thus far, most bioinspired examples represent a set of isolated inspirations from natural systems leading to non‐optimal CO_2_RR electrocatalysts with high activity and selectivity towards C_1_ products, but low current density in a CO_2_RR device (<≈10 mA cm^−2^ in an H‐cell) and long‐term stability (<120 h) substantially lower than targets for commercialization (>200 mA cm^−2^ over >8000 h at >90 % selectivity).[^3a^, ^38^, ^39^]

The adoption of NICE could lead to a more systematized bioinspired design strategy and accelerate the development of highly efficient bioinspired CO_2_RR electrocatalysts. The NICE methodology could become even more effective when paired with further advances in the fundamental understanding of the CO_2_RR mechanism. The complex interplay between the structure (nano‐ and meso‐scale) of the catalyst, its catalytic properties and durability, the electrolyte (pH, buffers), and the mass transport limitations is far from being completely understood.[^1^, ^3a^] Finally, synthesis protocols viable for mass production must be developed to enhance the low yield of electrocatalysts prepared by conventional approaches.

Apart from electrocatalysts, the commercialization of this technology is also contingent upon the significant improvement of the design of CO_2_RR devices to enhance their energy efficiency (i.e., high activity and selectivity at low overpotential). Mass transport limitations within the device must be resolved, while product separation and tolerance towards gas inlet purity must be improved.^[39]^ Nature provides examples that can be leveraged to circumvent these issues, through its intrinsically scaling hierarchical transport networks.

## Conflict of interest

The authors declare no conflict of interest.

6

## Biographical Information

*Panagiotis Trogadas is Assistant Research Professor in the Department of Chemical Engineering at UCL, working in the Centre for Nature‐Inspired Engineering. His research interests lie in the exploration of nature‐inspired electrochemical components, devices, and systems. He has won several research grants and delivered invited lectures and keynotes*.

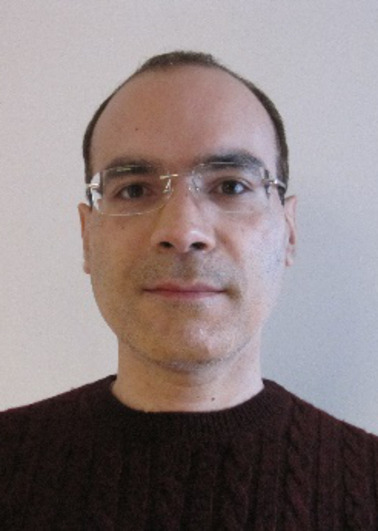

## Biographical Information

*Linlin Xu received her master's degree (2019) in Materialogy from Institute of Process Engineering, Chinese Academy of Sciences. She is currently a PhD student at University College London, working on nature‐inspired electrochemical devices, under the supervision of Prof. Marc‐Olivier Coppens, supported by UCL EPSRC DTP Studentships*.

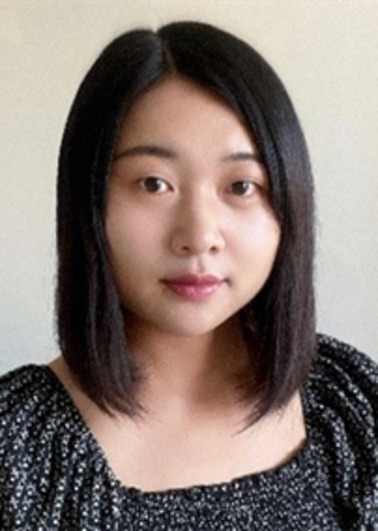

## Biographical Information

*Marc‐Olivier Coppens is Ramsay Memorial Professor in Chemical Engineering at UCL, since 2012, after professorships at Rensselaer and TUDelft. He is also Vice‐Dean for Engineering (Interdisciplinarity, Innovation). He directs the Centre for Nature‐Inspired Engineering, which was granted EPSRC “Frontier Engineering” (2013) and “Progression” (2019) Awards. He is most recognized for pioneering nature‐inspired chemical engineering (NICE). He is Fellow of RSC, IChemE, AIChE, Corresponding Member of the Saxon Academy of Sciences (Germany), Qiushi Professor at Zhejiang University, and has delivered >50 named lectures, plenaries and keynotes*.

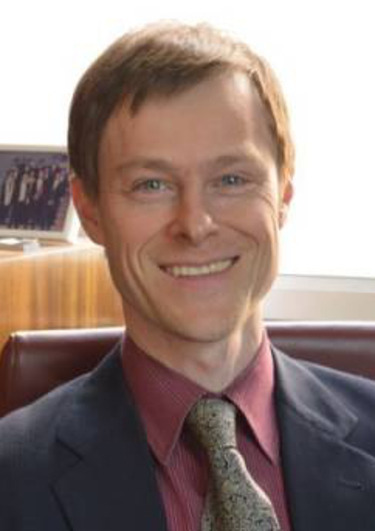

## Data Availability

Data sharing is not applicable to this article as no new data were created or analyzed in this study.
